# Use of Alternative Wood for the Ageing of Brandy de Jerez

**DOI:** 10.3390/foods9030250

**Published:** 2020-02-26

**Authors:** M. Valme García-Moreno, Manuel M. Sánchez-Guillén, María Ruiz de Mier, Manuel J. Delgado-González, M. Carmen Rodríguez-Dodero, Carmelo García-Barroso, Dominico A. Guillén-Sánchez

**Affiliations:** Departamento de Química Analítica, Facultad de Ciencias, Instituto Investigación Vitivinícola y Agroalimentaria (IVAGRO), Universidad de Cádiz. Campus Universitario de Puerto Real, 11510 Puerto Real, Cádiz, Spain; manuel.sanchez@uca.es (M.M.S.-G.); m.ruizdemier@gmail.com (M.R.d.M.); manuel.delgado@uca.es (M.J.D.-G.); maricarmen.dodero@uca.es (M.C.R.-D.); carmelo.garcia@uca.es (C.G.-B.)

**Keywords:** Brandy de Jerez, alternative woods, toasting wood

## Abstract

The use of alternative types of wood has arisen for the aging of the Brandy de Jerez, on a pilot plant level. In particular, besides the use of American oak, two more types of oak have been studied, French oak and Spanish oak, allowed by the Technical File for the ID Brandy de Jerez, and chestnut, which, though it is not officially allowed, is a type of wood which had been traditionally used in the area for the aging of wines and distillates. All of them have been studied with different toasting levels: Intense toasting and medium toasting. The study of the total phenolic composition (TPI), chromatic characteristics, organic acids, and sensory analysis have proven that chestnut leads to distillates with a higher amount of phenolic compounds and coloring intensity than oak. This behavior is the opposite as regards the toasting of the wood. Among the different types of oak, Spanish oak produces aged distillates with a higher phenolic composition and a higher color intensity. Regarding tasting, the best-assessed samples were those aged with chestnut, French oak, and American oak, and the assessors preferred those who had used a medium toasting level to those with an intense level.

## 1. Introduction

According to the Technical File of the Geographic Indication «Brandy de Jerez» [1], Brandy de Jerez is defined as a spirit with an alcoholic strength between 36% and 45% obtained from wine spirits and distillates aged in oak barrels of capacity under 1000l. The barrel must be previously seasoned with sherry wines prepared in the area located between the cities of Jerez de la Frontera, El Puerto de Santa María, and Sanlúcar de Barrameda (Cádiz, Spain), following the aging system of Criaderas and Solera in use in the Jerez region. Though these regulations just state that the barrels need to be made of oak, the most widely used wood in the area is the American oak, though occasionally, it is possible to find some brandies aged in French oak barrels.

During the aging process of spirits, physical-chemical processes where components of the distillates and compounds from the wood of the cask are involved take place [2,3,4,5]. They are mainly extraction phenomena, together with other chemical phenomena, as oxidation, esterification, Maillard reactions, polymerization, and polycondensation reactions. All of them affect in a way or another the final organoleptic characteristics of the aged distillates.

Due to this, the final characteristics of an aged distillate, as brandy, will depend on all factors which are involved in some way in the aging process [3,5], as the characteristics of the wooden barrel: Type of wood [6,7,8,9,10,11], volume of the barrel [12,13], toasting level of the wood [8,14,15,16,17], or aging time [18,19]; the conditions of the winery, and the technological conditions taking place during the aging process [13,20,21,22,23,24]. In Brandy de Jerez, these final organoleptic characteristics are also going to depend on the previous seasoning of the barrel [25].

Due to the current trend by the winemakers of offering increasingly more differentiated and particular products, it seems logical to use for that purpose those alternatives accepted by the Technical File of the Geographic Indication «Brandy de Jerez» [1], as the use of types of oak other than American oak, or, apart from them, to consider the use of other traditionally used wood types for the aging of distillates, as chestnut wood. There are some articles studying the use of other types of oak and of chestnut wood for the aging of wine distillates, in particular, of Portuguese brandies [7,8,19,26,27], and in cider brandy [13].

The purpose of this paper is the study of alternative wood types, on a pilot plant level, for the aging of Brandy de Jerez. To do so, besides the use of American oak (*Quercus alba*), two more types of oak have been taken into account, French oak (*Quercus robus*) and Spanish oak (*Quercus pyrenaica*), allowed by the Technical File of the Geographic Indication Brandy de Jerez, and chestnut (*Castanea sativa*). The latter, though not officially allowed, is a type of wood which has been traditionally used in the area for the aging of wines and distillates. All of them have been studied with two different toasting levels: Intense toasting and medium toasting. 

## 2. Material and Methods

### 2.1. Samples

The study was carried out in 16 l. wooden barrels (Tonelería J.L. Martínez Montilla, Spain) made from four different types of wood: American oak (AO) (*Quercus alba*), French oak (FO) (*Quercus robus*), Spanish oak (SO) (*Quercus pyrenaica*), and chestnut (CH) (*Castanea sativa*). Each group consisted of four barrels, two of them with an intense toasting level (IT) and the remaining two, of a medium toasting (MT). The difference between both toasting levels depends on the time which the barrel spends in the toasting process at 130–140 °C in an oven. For the medium toasting, the barrel stays in the oven for 5–7 min, whereas, for the intense one, it is kept for 10–12 min, allowing it to burn for 15–20 s. The barrels were previously seasoned for two months with Oloroso wine in an intermediate or *sobretabla* (over-table) stage, with less than a year old. This wine is a white wine oxidized typical of Sherry area. In the *sobretabla* state, the wine has not “touched” wood yet. That is, it has not aged in any type of wooden barrel; it has oxidized in the air. 

After those two months, the barrels were emptied and were then filled with the distillate to be aged. In this case, a wine distillate with an ABV of 65%, from the Airen variety, was used. The wine distillate was supplied by the González Byass winery, and it was obtained by column distillation.

The sampling was carried out six weeks afterwards, taking 100 mL of the sample and refilling with the corresponding non-aged distillate. 

### 2.2. Reagents

For total polyphenol index (TPI) determination, Folin–Ciocalteu reagent was purchased from Merck (Darmstadt, Germany) and anhydrous sodium carbonate was supplied by Panreac (Barcelona, Spain). 

Folin–Ciocalteu reagent, anhydrous sodium carbonate, and gallic acid, for total polyphenol index (TPI) determination, were purchased from Merck (Darmstadt, Germany), Panreac (Barcelona, Spain) and Sigma-Aldrich (Saint Louis, MO, USA), respectively. 

For organic acid analyses, ethylenediaminetetraacetic acid (EDTA), 2,2-bis(hydroxymethyl)-2,2′,2″-nitrilotriethanol (Bis-Tris), and trifluoroacetic acid (TFA) were purchased from Sigma-Aldrich (Saint Louis, MO, USA). Standards for calibration were supplied by Panreac (Barcelona, Spain), Sigma-Aldrich (Saint Louis, MO, USA) and Scharlab (Barcelona, Spain). 

UHPLC grade acetonitrile from Panreac (Barcelona, Spain), acetic acid from Merck (Darmstadt Germany) were used to prepare the UHPLC phases. Standards for calibration were purchased from Sigma-Aldrich (Saint Louis, MO, USA). Deionized water from EMD Millipore (Bedford, MA, USA) was used to prepare the chromatography phases, reagents and standards for calibration.

### 2.3. Enological Control Parameters 

A pH-matic 23 automatic titrator (Crison Instruments SA, Barcelona, Spain) was used to measure the pH and total acidity. A D.E. 2000 distiller extraction system (Laboratoires Dujardin-Salleron, Arcueil, France) was used for alcoholic proof measurements, following the official method from the International Organization of Vine and Wine (OIV). The alcoholic proof was then measured from the density of the distillate in an DMA 4500 M densimeter (Anton Paar, Ashland, OR, USA).

### 2.4. Phenolic Compounds and Furfurals

Phenolic compounds and furfurals were quantified by UHPLC. 13 phenolic compounds (gallic acid, protocatechuic acid, protocatechuic aldehyde, p-hydroxybenzoic acid, p-hydroxybenzaldehyde, caffeic acid, p-coumaric acid, vanillic acid, vanillin, syringic acid, syringaldehyde, sinapaldehyde and coniferaldehyde) and 3 aldehydes furanics (furfural, 5-methylfurfural and 5-hydroxymethylfurfural) were identified. The system used was a Waters Acquity UPLC with a PDA detector and the column was an Acquity UPLC C18 BEH, 100 × 2.1 mm (i.d.) with 1.7 µm particle size (Waters Corp., Milford, MA, USA). Two eluents were used: A phase that consisted of 3% acetonitrile, 2% acetic acid and 95% ultrapure water, and B phase that consisted of 85% acetonitrile, 2% acetic acid, and 13% ultrapure water. A flow rate of 0.7 mL/min and 47 °C of column temperature was applied, the injection volume was 2.5 µL. The gradient used was as follows: 0 min, 100% A; 3 min, 90% A (curve 6); 4 min, 90% A; 6.5 min, 25% A (curve 6). Finally, the column was washed with 100% B for 3 min and equilibrated with 100% A for 3 min. Samples and standards were filtered through 0.22 µm nylon membranes, and they were injected in duplicate [28]. The detection by UV absorption was conducting by scanning between 250 and 400 nm, with a resolution of 1.2 nm. The identification of each compound was carried out by comparing retention times and UV–Vis spectra of the peaks in distillates with those previously obtained by the injection of standards. The results were expressed in mg of compound per liter of spirit.

### 2.5. Total Polyphenol Index (TPI)

A Thermo Helios Gamma UV-VIS Scanning spectrophotometer (Thermo-Fisher Scientific, Waltham, MA, USA) was used to determine TPI [29]. The instrument was calibrated with gallic acid aliquots in the range 10–1000 mg/kg. Samples measurements were carried out in duplicate. The results are expressed in mg gallic acid equivalent (GAE) per liter of sample.

### 2.6. Color Measurements

Color measurements were made with a Thermo Helios Gamma UV-VIS Scanning spectrophotometer (Thermo-Fisher Scientific, Waltham, MA, USA) in the range of 380–780 nm. A Microsoft Excel 2016 (Microsoft Corp., Redmond, WA, USA) data sheet was used to convert the spectra into CIELab parameters, following the method of Commission Internationale de l’Eclairage (CIE) [30]. Each sample was measured in duplicate. 

### 2.7. Short Chain Organic Acids

Short chain organic acids (citric, tartaric, malic, succinic, lactic, formic and acetic acids) were quantified by an isocratic HPLC system with conductivity detection [31]. The system consisted of two Waters 515 HPLC pumps, a Waters 717 autosampler (Waters Corp., Milford, MA, USA), an LBK chromatography oven model 2155 (Pharmacia, Sweden), and a Milton Roy Conductomonitor III conductivity detector (LDC, FL, USA). The column was an exclusion column Phenomenex Rezex ROA-Organic AcidH + 8%, 300 (l) × 7.8 mm (i.d.) (Phenomenex, Torrance, CA, USA). The mobile phase consisted of 2.5 mM solution of tri-fluoroacetic acid (TFA; 0.4 mL min^−1^). A solution consisted of 2.5 mM TFA, 20 mM bis-[2-hydroxyethyl]imino-tris-[hydroxy-methyl]methane (bis-tris buffer), and 100 mM EDTA was added at the column out-let to increase the detection sensitivity. A flow rate of 0.4 mL/min and 60 °C of column temperature was applied, the injection volume was 40 µL. Samples and standards were filtered through 0.45 µm nylon membranes, and analyses were carried out in duplicate. The identification of each compound was carried out by comparing retention times of the peaks in distillates with those previously obtained by the injection of standards. The results were expressed in mg of compound per liter of spirit.

### 2.8. Sensory Analysis 

All sensory analyses were carried out in a normalized tasting room [32]. The temperature was set at 22 °C. 15 mL of each sample after two months in barrel was presented in a standard wine glass [33]. The cups were covered to avoid the loss of aromas and codified with three-digital numbers. 

The panel was formed by seven judges (four women and three men, age ranged from 25 to 50 years) who belong all of them to the laboratory personnel. They were submitted to a three-sessions training on the general and specific organoleptic qualities of ageing distillate. A duplicated descriptive profile of two distillate samples was employed to validate the judges’ reproducibility; whereas, for each descriptor, a two factor ANOVA (judges × samples) of the descriptive data allowed the homogeneity of the panel to be evaluated. Regarding this, in a few cases where standard deviations were higher than 2 the divergent values were eliminated. 

An evaluation sheet was prepared with all those descriptors that had shown a higher descriptive ability in a preliminary test in which several distillate samples were shown to the judges. The selected descriptors were, in the visual phase, color intensity and color impression; in the olfactory phase: Oxidative odor, nuts, toasted, woody, aromatic intensity and odor impression; and finally, in the gustative phase: Alcoholic, bitter, persistence, body and gustative impression.

Two evaluation sessions were necessary to study the samples and the duplicates of two (for judges’ reproducibility). In each session, five cups (four samples and one duplicate) were presented to each judge. The evaluation was made within the following scale: For descriptor intensity (0—not present; 2—slight; 4—medium; 6—strong; 8—very strong); for general impression (0—bad; 2—mediocre; 4—acceptable; 6—good; 8—very good) [34].

### 2.9. Statistical Analysis

The Statgraphics 18 software package (Statgraphics Technologies, Inc., The Plains, VA, USA) was employed for ANOVA, principal component analysis (PCA), linear discriminant analysis (LDA) and cluster analysis (CA). Microsoft Excel 2016 (Microsoft Corp., Redmond, WA, USA) was employed for other statistical parameters.

## 3. Results and Discussion 

### 3.1. Enological Control Parameters 

Table 1 shows the data for pH, total acidity, and alcoholic degree (% Alcohol by volume, ABV) of the studied samples. At the end of the study, the aged samples showed higher total acidity values than the initial distillate samples, as can be expected for this type of aged drink [35]. The pH of the samples is around 4, which is characteristic of new brandies [35]. Lastly, the alcoholic strength of all studied samples is around 60% ABV, being slightly lower in the aged samples than in the initial ones. This decrease is more significant in those samples which were aged in CH wood, though it cannot be considered in any case that the difference is a significant one for a significance level of α = 0.05 (HDS Tuckey’s Test). 

No significant differences are observed either regarding the toasting level for the same type of wood in any of the three oenological parameters considered. However, a given dependence on the toasting level of the barrel can be observed for total acidity: Those samples belonging to distillates aged in medium toasting oak barrels show slightly lower values than those aged in intense toasting oak barrels. For those distillates aged in chestnut barrels, the behavior is the opposite: The average total acidity value slightly decreases with the toasting level of the wood.

### 3.2. Phenolic Composition of the Aged Spirits

The content in low molecular weight phenolic compounds determined by means of UHPLC in the distillates obtained after a six week aging period, expressed in mg per liter of distillate, is shown in Table 2. Levels around mg/L can be seen in the concentration of gallic acid, p-hydroxybenzoic acid, vanillic acid, syringic and caffeic acid, aldehydes, vanillin, syringaldehyde, sinapaldehyde and coniferaldehyde, and furanics, furfural, 5-methylfurfural (MF), and 5-hydroxymethylfurfural (HMF). All these compounds are easily found in samples of brandy aged in wood [5,6,9,17,20,25,28,36,37], except for caffeic acid [21]. In general, the values of the phenolic compounds of the distillates aged in this study present values which are over the shown ones for Brandy de Jerez samples with a longer aging time [21,25,28]. This highlights the relevance of the size of the barrel during the extraction process, in particular, the surface/volume ratio, and as a result, a higher concentration in the distillates for the same aging time [38]. We could think that these values are due to the fact that the studied samples are taken at 6-week aging, and due to this, the extraction process is in its first stage [39]. However, it is important to remember that the barrels used for this study have been previously seasoned with Oloroso wine; i.e., before having the distillate, they contained wine, and as a result, the high extraction stage already took place during the previous seasoning stage.

Gallic acid is the low molecular weight phenolic compound, quantified in the aged samples, which presents higher concentration volumes. Those distillates aged in CH present the highest values—28.33 mg/L the samples from the CH-MT barrels and 24.03 mg/L those from CH-IT barrels. The samples which were aged in oak barrels showed lower values, between 2 and 5 mg/L of gallic acid. Three groups could be distinguished, according to the Tukey’s HSD test (0.05). The first one would be formed by the samples from SO (SO-MT and SO-IT) barrels with those from the FO-MT ones. The following group would include samples from FO (FO-MT and FO-IT) barrels with SO-IT. The last group would consist of the samples which were aged in AO (AO-MT and AO-IT) barrels. This same behavior as regards the gallic acid from the oak and chestnut wood has been observed by other authors during the aging process of distillates [6,40,41], and in hydroalcoholic extracts [17]. In fact, a characteristic feature of drinks aged in chestnut wood is their high concentration of gallic acid [5,9,36,42]. The toasting level of the wood of the barrel has a significant effect on the concentration of gallic acid for those distillates aged in CH, and thus, those distillates from CH-MT barrels show a higher concentration than those aged in CH-IT ones, as has been previously mentioned. These data also agree with those found by other authors for Portuguese brandies aged in chestnut barrels [17,26]. As regards those distillates aged in oak barrels, no significant effect of the toasting level of the wood as regards gallic acid concentration can be observed, similar to the findings of Chatonet et al. [43] also for oak wood. However, there are some authors who have noted an increase in the concentration of gallic acid related to the toasting level in chestnut chip extracts [15], and in synthetic wine aged with oak and chestnut chips [10,17]. Other authors have discovered a decrease of such component in brandies aged in chestnut barrels [26], and in chestnut chip extracts [16]. The reason for this uneven behavior of the gallic acid levels as regards the toasting level of the wood can be found in the own nature of gallic acid. This acid is a highly reactive molecule which is prone to oxidation [43]. Due to this, it can be involved in different oxidation and polymerization reactions, which make its behavior change from one study to the other.

The samples from distillates aged in CH present higher values of p-hydroxybenzoic, vanillic, and syringic acids to those aged in oak barrels, being this effect higher for the p-hydroxybenzoic and syringic acids. The concentrations found in the samples which had been aged for six weeks are approximately half of those found by Canas et al. [26] for brandies aged for two years in chestnut barrels. The concentration of these compounds in the studied samples as regards the type of studied wood presents a similar behavior to that observed for gallic acid: A significant difference between those distillates from oaks and the distillates coming from chestnut is found. The samples aged in FO show the lowest levels and those from CH, the highest ones. As regards the toasting level, oak barrels with an intense toasting level produce aged distillates with slightly higher phenolic acid levels [5], whereas, those aged in chestnut barrels show opposite behavior. This increase is not significant for any of the four studied types of wood, in line with the findings of other works in the bibliography [15]. According to Cernisev [44], taking into account the syringic and vanillic acid levels, the CH samples would be in the old or extra old brandy group, whereas, those aged in oak would be in the old or moderate brandy group, proving again the smaller barrel size issue mentioned in this study.

Regarding caffeic acid, it has just been able to be quantified in our study in those distillates aged in AO, showing levels of around 0.60 mg/L. Those distillates aged in CH showed values below the quantification limits, and the distillates aged in SO and FO showed levels below the detection limits. In the detected cases, no significant differences were found so as to the toasting level of the wood. As regards the presence of this acid in the brandy samples, we found authors in the bibliography who thought that the presence of this acid in Brandy de Jerez samples is due to the wine previously contained in the barrel during the seasoning stage [21]. During this stage, there is an exchange between the barrel and the wine contained in it. On the one hand, there is a blockage of the pores of the barrel with compounds from the wine, and on the other, there are also precipitation and co-precipitation processes with tartrates, which triggers a quick dissolution of the precipitated compounds during the aging stage of that distillate in such barrel. These authors found the content in caffeic and/or p-coumaric acids in Brandy de Jerez (and other Spanish brandies which are also made from seasoned barrels) as a differentiating feature of these spirits as compared to other distillates. Other authors have found significant amounts of this acid in toasted and natural oak wood extracts, with a synthetic wine dissolution with 12% ethanol [45]. These authors found that the concentration of caffeic acid in the extract samples is significantly affected by the type of oak studied and by the level of toasting of the wood: Toasting produces a decrease in the concentration, and the AO extract shows slightly higher values to those aged in FO. 

All of the samples of the studied aged distillates show a significant amount of phenolic aldehydes extracted from the wood: Vanillin, syringaldehyde, coniferaldehyde, and sinapaldehyde. It is well known from long ago that these components are present in oak wood, both independently and joined to other components [46,47]. The processes which take place during the aging of the brandies in wood barrels (ethanolysis, extraction) are responsible for their appearance in aged brandies [47,48]. Of the four found aldehydes, the most common in all studied samples is sinapaldehyde, as happened in the studies of Sanz et al. [16] for chestnut wood. The samples aged in FO (FO-MT and FO-IT) show the lowest values, whereas, those aged in CH (CH-MT and CH-IT) show the highest ones, except for syringaldehyde, where those samples aged in AO-IT and CH-MT show the highest values. SO obtains aged distillates with high phenolic aldehyde levels too, statistically similar to those of CH, but unlike AO or FO, whose values are statistically lower [16] (Table 2). The samples aged in CH-MT are the ones which show, again, the highest values, and those aged in FO-MT, the lowest ones. This behavior is similar to the one found for phenolic acids (except for gallic acid) when observed by other authors regarding the concentration values found in the distillates aged in FO and CH [36]. In our study, it is not possible to use these components as markers for the type of wood used in the aging, as happens in the research made by Canas et al. [6] with Portuguese brandies. Taking into account the toasting level of the wood, we can see that the concentration values decrease slightly with the intensity of the toasting in the samples aged in CH and increase in most of the samples aged in oak. This behavior is the opposite to the one found by Sanz et al. and other authors [5,26,43]. The increase of the levels of these compounds with the toasting of the oak barrels can be, due to the fact that the thermal treatment of the wood which takes place during the manufacturing of the barrel provokes a thermal degradation of the lignin and an increase of the permeability of the wood, increasing the content of these components in the distillates aged in them [11,16,24,36]. 

In all of the studied aged distillates, detectable and quantifiable amounts of three furanic aldehydes have been found: F, MF, and HMF. Low levels of F (0.45 mg/L) have been detected in the initial distillate sample, but no detectable levels of MF or HMF have been found. The concentrations of F and HMF are significantly higher than those of MF in all of the studied aged distillates. The samples which had been aged in CH showed the highest values, followed by those aged in SO, AO, and lastly, FO barrels. The origin of these components in the wood is related to the carbohydrates of the wall of the plant cells of the wood. The thermal degradation of the glucose of cellulose leads to the development of HMF and MF, and the thermal degradation of pentoses, main components of hemicelluloses, to the development of F. As hemicelluloses are the most sensitive polymers to heat in wood, their degradation during the heat treatment underwent by the wood for the manufacturing of the barrel favors the development of furfural as the main furanic aldehyde in toasted oak and chestnut woods [26,43,49]. Chestnut wood presents a higher amount of cellulose than oak wood [26], which might be the reason for the higher levels of these compounds found in those distillates aged in CH wood. Regarding the toasting level of the wood, all three components show a similar behavior among them and similar as well to that of phenolic aldehydes: Their concentration increases as the toasting level of oak wood increases, more specifically in AO and FO samples, as in SO samples, no significant differences are found regarding the toasting level, whereas, in CH samples, they decrease with the toasting level. We can find in the certain bibliography controversy as regards the effect of the toasting level of the wood on the concentration of these furanic aldehydes. Thus, Chatonnet et al. [43] observed the highest values in medium toasting woods, whereas, other authors found otherwise [26]. Chatonnet et al. [43] suggest that the decrease of the level of furan derivatives with intense toasting levels can be a consequence of the degradation and volatilization of the components, due to the high temperatures of the toasting process [14]. In this sense, Soares et al. [17] state that the different behavior across different studies might be due to the fact that there is no clear correspondence between the toasting level and the temperature at which the toasting takes place.

In general, we can say that brandies aged in chestnut wood show higher levels of low molecular weight phenolic compounds than those aged in oak barrels. Among the different types of oak woods, aged brandies show low molecular weight phenolic levels according to the sequence FO <AO <SO, though this order is not met for all studied components. As regards the toasting level, the brandies aged in chestnut wood with an intense toasting level show lower levels than those aged in chestnut wood with a medium toasting level. In the case of the three studied oak types, this behavior is reversed. 

### 3.3. Total Phenolic Index

The TPI data of the studied samples, expressed in mg of equivalent gallic acid per liter of distillate, are also shown in Table 2. Taking into account that the initial distillate shows values under the detection limit of the method, the results obtained for the aged samples are exclusively, due to the interaction between the distillate and the wood of the barrel during the aging time. As can be seen in the obtained data, a clear difference arises between those samples aged in CH and those aged in oak. This result is coherent with the data shown for low molecular weight phenolic compounds: Chestnut barrels produce distillates with a higher concentration of phenolic compounds than oak barrels. As has been mentioned in detail in the previous paragraph, this wood transfers a larger amount of phenolic compounds during the aging processes of the distillates, leading to aged distillates with higher phenolic content. In second place, SO is found, whereas, FO and AO transfer similar amounts, not being possible to significantly determine this amount in those distillates aged in oak based on TPI. Alañón et al. [41] obtained similar results as regards the behavior of AO and SO woods in methanol extracts: *Quercus alba* (AO) extracts show a lower TPI than *Quercus pyrenaica* (SO). However, this is not so for *Quercus robus* (FO) extracts, which present a reverse behavior to the one found in our study. Fernández de Simón et al. [50] observed that the content in total phenols is higher in *Quercus pyrenaica* than in *Quercus robus*, determined in methanol/water (1:1) extracts [41]. Other authors also observed that chestnut wood presents a more significant assignment of phenolic compounds that oak wood [10]. 

As regards the toasting level of the wood, the TPI values of the studied samples follow an expected pattern: They increase as the toasting level of the wood increases for oaks and just the opposite for chestnut, in line with the findings of Sanz et al. [16]. These differences are significant for two of the four types of wood, FO and CH, if we take into account the data expressed by liter of distillate, but there are no significant differences in any distillate aged in any of the types of oak if we consider the data per liter of absolute alcohol (Table S1). This behavior is similar to the one observed by Sanz et al. [16], who discovered a decrease of IPT in chestnut chips as they were toasted. However, other authors [17] observed a systematic decrease of IPT with the toasting level in samples of synthetic wines aged with oak and chestnut chips. The high reactivity of the phenolic compounds, together with their lability to heat and the non-homogenization of the burning criteria of the wood has already been mentioned in this paper. This is the reason why it is possible to obtain such different results in the bibliography as regards the phenolic compound levels in aged distillates in comparison with the toasting level of the wood.

### 3.4. Chromatic Characteristics

The study of the color of the sample (Table 3) showed expected values: All aged samples showed color differences as compared to the initial distillate (parameter ΔE_00_). Those distillates aged in FO and AO showed a lower increase in color with aging, and the samples aged in SO and CH, a higher color increase, with ΔE_00_ values over 25 units. In particular, the largest color increase was shown by those distillates aged in chestnut wood barrels with a medium toasting level, with ΔE_00_ levels around 36 units. This increase in the color is clearly related to the aging process in the wood barrel [23,36,44,51], and to the extraction and oxidation reactions which take place between the compounds extracted from the wood and those of the distillate. This result agrees with the data for phenolic compounds and IPT of the distillates aged in this type of wood. 

The aged distillates showed a decrease in brightness (L*) as compared to the initial distillate. This is more marked in the samples aged in CH barrels as compared to those aged in oak barrels, according to the increase of the ΔE_00_ parameter. 

As regards parameter a* (green to red tones), those distillates aged in FO and AO showed negative values close to zero, i.e., they showed more greenish tones than the distillates aged in CH and EO, which presented more reddish tones, positives values of parameter a*. The distillates aged in chestnut wood presented the highest values of this parameter. Parameter b* (blue to yellow tones) increases with aging in all trials (yellow tone increases). The distillates aged in CH are the ones which present the highest values.

According to Canas et al. [36], the increase of yellow tones can be related to the oxidation of ellagitannins of the wood [52], with the condensation reactions among tannins in the presence of acetaldehyde and phenolic aldehydes, as happens with the aging of wines in wood [53] or with the development of melanoidins and other colored components during the aging process [44,54].

### 3.5. Organic Acids

The aged distillates present significant amounts of succinic, lactic, and acetic acids (Table 4), unlike the initial distillate, where just lactic and acetic acids were able to be quantified. These two acids are frequent in aged distillates, as they come from the initial distillate, due to the fact that they are volatile acids which can pass from the wine to the distillate during the distillation process [37], as happens in our case. Besides, acetic acid presents another different origin to the distillation process: It is known that it is a secondary product of the heat degradation of the wood. It is easily detected by its smell during the manufacturing process of the barrels, in particular, after the barrels are sprayed with water when exposed to a heat source [43,55]. Succinic acid, on the contrary, is not a volatile acid and not characteristic of distillates. Its detection and quantification in the samples of aged distillates of our study are due to the previous seasoning process of the barrels which have been used for our study. There are several studies proving the presence of non-volatile organic acids (tartaric, malic, succinic...) in Brandy de Jerez [25,31,37,56,57], due to the use of barrels which had been previously seasoned with Sherry wine, as is the case of the barrels used for this study. 

The quantified levels of succinic acid in the aged samples of our study are slightly higher in the samples aged in intense toasting barrels than in those aged in medium toasting barrels, though the differences are not significant for a = 0.05 (Tukey’s HSD) in most of the used woods. No significant differences can be observed either among the different types of wood, as the distillates show very similar values. The fact that the values obtained for this acid are higher than those obtained by Moreno et al. [56] for Brandy de Jerez, and by those obtained by Guillén-Sánchez et al. [25] for samples of brandies aged in barrels which had been previously seasoned with Oloroso wine, as in our case, is worth being mentioned.

As regards the lactic acid levels, some differences can be observed depending on the type of wood of the barrel. In this way, the SO and CH barrels show similar values to those of the initial distillate sample (181.6 mg/L). On the other hand, those aged in AO and FO show higher values, between 333.5 mg/L and 403.8 mg/L. No direct relationship is observed either for this acid between its concentration and the toasting level of the wood. These levels of lactic acid are much higher than those found by Moreno et al. [57] in brandy samples of the three commercial categories (*Solera*, *Solera Reserva*, and *Solera Gran Reserva*), although they are similar to those found by other authors in Brandy de Jerez samples [25,37], being the lowest ones more typical of *Solera* brandies and the highest ones, of *Solera Gran Reserva* brandies [37]. 

The detected levels of acetic acid in the aged distillates are higher than those detected in the initial distillate, approximately double the amount, being the distillate aged in SO-MT the one with the lowest concentration (210.1 mg/L), and the sample aged in AO-IT, the one showing the highest concentration (307.6 mg/L). No clear trend is observed as regards the origin of the wood, but there is one regarding the toasting level: Slightly higher values are shown by the samples which were aged in wood with an intense toasting level than in those with a medium toasting level for oaks, and just the opposite for CH. Nevertheless, this difference cannot be considered to be significant in any case (α = 0.05, Tukey’s HSD). They found levels are similar to those found by other authors in Brandy de Jerez samples [25,37,57].

### 3.6. Global Chemical-Physical Assessment of the Aged Distillates

**Figure 1:**
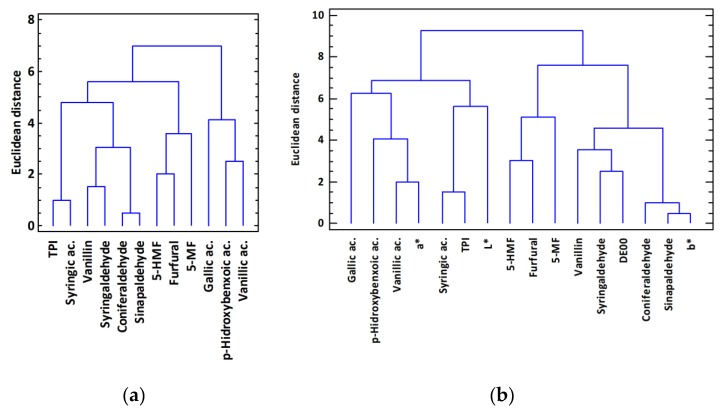
Cluster analysis obtained using the Euclidean distance as metrics and the Ward method as clustering rule for the standardized variables. (**a**) Phenolic content. (**b**) Phenolic content and chromatic characteristics.

To globally analyze the obtained results, a study of the studied variables was carried out by means of a cluster analysis, so as to determine which variables behaved similarly in the studied samples. Figure 1a shows the obtained cluster using the Euclidean distance as metrics and the Ward method as clustering rule for the standardized variables related to the phenolic content. It shows a clear distinction into four clusters of variables: Phenolic aldehydes, furanic aldehydes, phenolic acids, and TPI together with syringic acid. This proves that there is a relationship between the behavior of the variables during aging and the chemical nature of such aging. If the chromatic variables are included in the study (Figure 1b), we can observe that component a* is related to the content in phenolic acids (vanillic ac, p-hydroxybenzoic ac., and gallic ac.), component b* and ΔE_00_, with the content in phenolic aldehydes, and L* with TPI and syringic acid. This indicates a clear correlation between the chromatic variables and the content in phenolic compounds in the samples.

**Figure 2:**
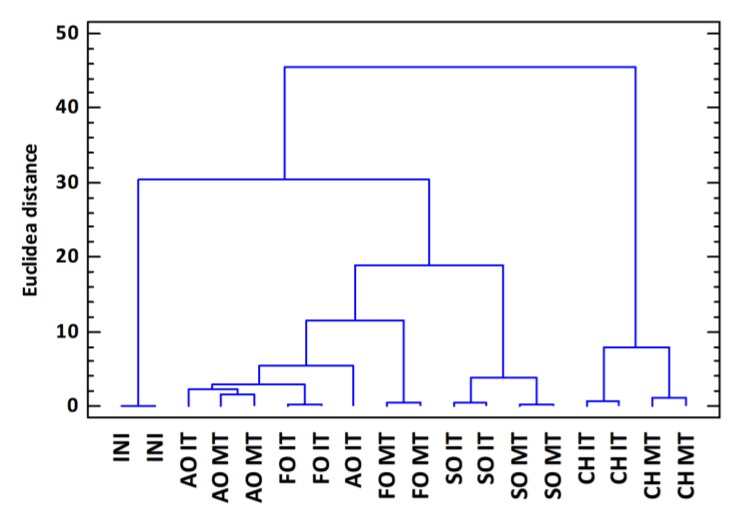
Cluster analysis obtained using the Euclidean distance as metrics and the Ward method as clustering rule for the analyzed samples using as variables the phenolic compounds, the TPI, and the chromatic variables (INI: Initial distillate; AO: American oak; FO: French oak; SO: Spanish oak; CH: Chestnut; IT: Intense toasting level; MT: Medium toasting level).

If this same statistical study is carried out for the analyzed samples using as variables the phenolic compounds, the TPI, and the chromatic variables we obtain the cluster, shown in Figure 2. In this case, the samples are grouped into three large groups: The initial distillate samples, the samples aged in CH, and those aged in oak. In the last group, samples are also grouped into two subgroups: The distillates aged in SO and those aged in FO and AO. This study proves the behavior which has been observed throughout the whole study: Chestnut barrels have behaved differently to those made of oak as regards the levels of phenols in the aged distillates. Similarly, within the types of oak, Spanish oak has been the one which has led to the most different aged distillates as compared to the other two oak types, whereas, FO and AO led to aged distillates with more similar chemical characteristics, being the FO-MT samples the ones which present more differences within the group. 

**Figure 3:**
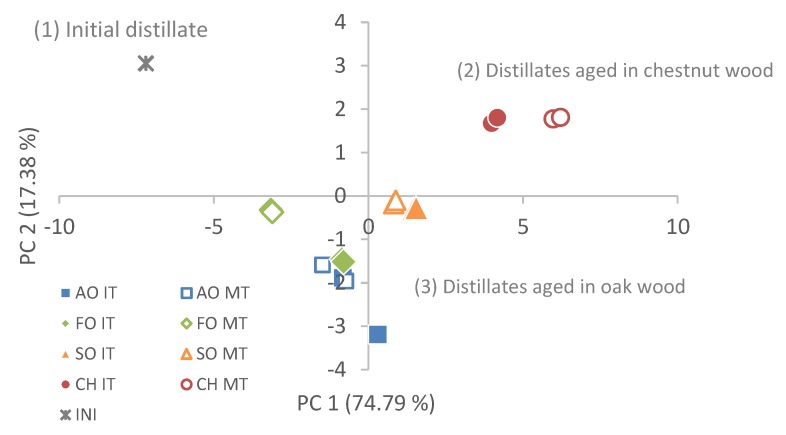
Biplots of vector loadings and distribution of samples for Principal Component analysis (PCA) according to the type of barrel and toast used. PC1 versus PC2.

If a Principal Component Analysis (PCA) is carried out taking into account the phenolic compounds, the TPI, and the content in organic acids, three main components explain 97.8% of the total accumulated variability: PC1 explains 74.8% of the variability of the data, PC2, 17.4%, and PC3, 5.6%. In Table 5, the weights of the variables for each component are shown. The scatter plot of the samples consisting of PC1 and PC2 is shown in Figure 3. The samples can once again be grouped into three groups: (1) The initial distillate, (2) the distillates aged in chestnut wood, and (3) the samples aged in oak wood. Within this last group, those samples aged in SO form their own group again, those aged in FO-MT form another subgroup, and the last subgroup consists of those aged in AO and FO-IT. 

On a chemical level, we can clearly see that oak barrels lead to distillates with a different chemical composition to those aged in chestnut, being SO the most different one among the oak types, showing a behavior closer to CH, whereas, AO and FO develop more similar distillates. Of all them, FO-MT produces the distillates with the lowest level of extractable compounds and with a lesser color.

### 3.7. Sensory Analysis

The data obtained in the sensory analyses carried out on the aged distillates were filtered by means of the Grubb and Kolmogorov-Smirnov tests. In the first place, a Grubb test was implemented in order to remove outliers. Then, the normality of the distribution of the data was checked through the Kolmogorov-Smirnov test. Once the normality of the sensory data had been checked, the study of the variance of the data was implemented, observing that some of the descriptors presented significant differences (*p* < 0.05). 

**Figure 4:**
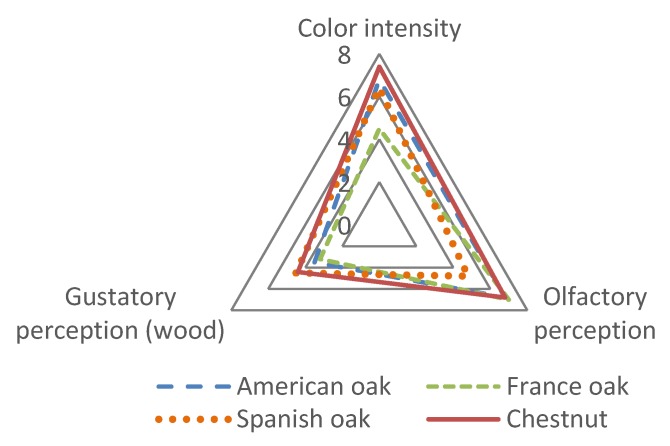
Color intensity, gustatory perception (wood) and olfactory perception impressions in the sensorial analysis of distillates aged in American oak, France oak, Spanish oak and Chestnut.

As regards the type of wood, the visual perception (color intensity), the olfactory perception, and the gustatory perception were assessed. As regards visual perception, the assessors considered that those distillates aged in CH presented the highest color intensity and were also the best valued ones. The samples aged in FO barrels presented, on the other hand, the lowest color intensity. This result agrees with the data obtained for the ciede2000 parameter, as has been already mentioned in Section 3.4*. Chromatic characteristics*. As regards the gustatory perception, the assessors perceived a certain sweet taste, with vanilla and wood notes. The distillates aged in CH and SO were the ones which presented the highest scores in the gustatory perception, especially as regards the wood note. In relation to the olfactory perception, the best valued samples were those aged in CH, AO, and FO, presenting similar scores and higher than those for SO. Those distillates aged in SO did not present a highly positive olfactory perception despite the fact that they are the ones which, together with CH, presented the highest intensity in aroma (Figure 4). 

As regards the toasting level, no significant differences can be found in the notes of the assessors for this parameter. However, the samples from the medium toasting level woods were better valued than those from intense toasting barrels, as the former had a sweeter gustatory perception and the assessors liked them best. 

These results show how FO and CH are types of wood which are very close to the level of AO for the aging of Brandy de Jerez, being SO the worst one according to the opinions of the assessors. Similar results for the aging of distillates in chestnut wood were already observed by other authors [5].

## 4. Conclusions

The study, at the pilot plant level, of alternatives wood for the aging of the Jerez brandy, showed that there are differences in the distillates aged according to the wood used, and in particular between the chestnut wood and the oak wood. In addition, differences have also been found according to the degree of toasting of the barrel wood.

In general, distillates aged in chestnut wood presented higher levels of phenolic, furfural and IPT compounds than distillates aged in oak barrels. Among the oaks, the distillates aged in SO were those that presented the highest values, and those of FO those that presented lower levels, although this order is not the same in all the compounds studied. This effect is especially significant in the concentration of gallic acid: Distillates aged in oaks presented concentration values of gallic acid between 2 and 10 times higher than those aged in oaks. Among these, the SO was the wood that gave more gallic acid, followed by the FO and finally the AO. In the case of phenolic aldehydes, the samples from CH are once again the ones that presented the highest values, although SO resulted in aging with levels of phenolic aldehydes statistically similar to those of CH.

As for the degree of toasting of the wood, the barrels of CH-MT resulted in distillates with a higher concentration of phenols, furfurals and IPT than those of IT. In the oaks this behavior is inverse; it is the barrels of IT that gave rise to distillates with higher concentrations of this type of compounds. However, it is noteworthy that the ratio of the concentration of gallic acid with the degree of toasting in aged distillates is only observed in aged samples in chestnut wood, not in oak samples.

The concentration values of phenolic compounds found in these aged distillates are higher than those reported by other authors for Jerez brandies with a longer aging time. This fact is explained by the smaller size of the barrel used in the study, 16 L of capacity compared to 600 L of the usual barrels for the aging of the Jerez brandies. This implies a greater surface/volume ratio, and therefore, a greater extraction for the same sample volume, resulting in aged distillates with higher concentration levels of compounds extracted for the same aging time.

Regarding the color of the aged samples, those from FO and AO are the ones that presented a smaller increase in color with aging, and those from SO and CH were the ones that showed a greater increase in color. Specifically, the most colored samples were those aged in chestnut barrels with medium roasting, the result being consistent with the data of phenolic compounds and IPT of distillates aged in this type of wood. Distillates aged in FO and AO showed more greenish tones than distillates aged in CH and EO, which presented more reddish tones.

Aged distillates also have significant amounts of organic acids: Succinic, lactic and acetic acids. Taking into account that succinic acid is not a characteristic acid of distilled beverages, its presence in these distillates is due to the previous process of wrapping that the barrels that have been used in this study have undergone. The concentration levels of succinic acid found in these samples are not affected by wood or toasting. The values obtained for this acid are higher than those found in the bibliography for brandies of Jerez.

The levels of lactic and acetic acids in aged distillates if they are affected by the wood and by the level of toasting, respectively. Distillates aged in SO and CH have similar levels of lactic acid to those in the initial distillate sample, and those aged in AO and FO have higher values. These levels are similar to those found in samples of Brandy de Jerez, being the lowest of brandies solera type and the highest of brandies solera grand reserve type. The levels of acetic acid found in aged distillates are similar to those found by other authors in samples of sherry brandies. These levels are slightly higher in oak samples aged in wood with intense toasting, in the chestnut samples the highest levels are found in those from medium toasting.

At the organoleptic level, the distillates of CH, FO and AO were very well valued by the judges, particularly those of medium toasting, not the SO distillates, since they were valued the worst.

The application of multivariate statistical techniques has revealed correlations between variables and samples. Thus, the cluster analysis showed a clear correlation between the chromatic variables and the content in phenolic compounds of the samples: It has been observed how the component a* of the color (shades of green to red) is related to the content of phenolic acids (vanillic ac, p-hydroxybenzoic ac and gallic ac.), the component b* (shades from blue to yellow) and ΔE_00_, with the content of phenolic aldehydes, and the L* with the TPI and the syringic acid.

Both the cluster analysis of samples and the PCA allowed grouping the samples into three large groups: The initial samples, distillates aged in CH and those aged in oaks. That is, chestnut wood distillates have a different chemical composition that allows them to differentiate from those aged in oak wood. Among the oaks, Spanish oak is what gives rise to more differentiated distillates, with the highest level of removable compounds. French and American oaks give rise to more similar distillates and with a lower level of extractable compounds, in particular, phenolic compounds. 

## Figures and Tables

**Table 1:** pH, total acidity (g/L Tartaric acid) and alcoholic degree (% Alcohol by Volume, ABV) of distillates ageing in barrel of American, French and Spanish oak, and Chestnut.

	Initial	American Oak		French Oak		Spanish Oak		Chestnut	
		Intense Toast	Medium Toast	Intense Toast	Medium Toast	Intense Toast	Medium Toast	Intense Toast	Medium Toast
pH	3.93 ± 0.02 ^b^	4.15 ± 0.01 ^f^	4.11 ± 0.01 ^e^	3.97 ± 0.00 ^c^	3.97 ± 0.00 ^c^	4.03 ± 0.01 ^d^	4.09 ± 0.01 ^e^	3.91 ± 0.00 ^b^	3.85 ± 0.01 ^a^
Total acidity	0.58 ± 0.02 ^a^	0.71 ± 0.05 ^a,b^	0.68 ± 0.07 ^a,b^	0.90 ± 0.05 ^c^	0.82 ± 0.03 ^b,c^	0.77 ± 0.02 ^b,c^	0.69 ± 0.05 ^a,b^	1.12 ± 0.05 ^d^	1.21 ± 0.05 ^d^
Alcoholic degree	61.97 ± 0.95 ^a^	56.25 ± 2.53 ^a^	55.04 ± 1.16 ^a^	59.31 ± 2.37 ^a^	60.42 ± 1.52 ^a^	57.45 ± 3.15 ^a^	57.46 ± 2.56 ^a^	55.48 ± 2.89 ^a^	55.28 ± 2.87 ^a^

Values in the same row with different letters are significantly different (*p* < 0.05).

**Table 2:** Phenolic compounds contents (mg/L) and total polyphenol index (TPI) (mg/L gallic acid equivalent) of distillates ageing in barrel of American, French and Spanish oak, and Chestnut.

	Initial	American Oak		French Oak		Spanish Oak		Chestnut	
		Intense Toast	Medium Toast	Intense Toast	Medium Toast	Intense Toast	Medium Toast	Intense Toast	Medium Toast
Gallic ac.	n.d.	4.88 ± 0.12 ^c^	4.60 ± 0.58 ^c^	3.04 ± 0.05 ^b^	2.78 ± 0.06 ^a,b^	2.85 ± 0.09 ^a,b^	2.00 ± 0.32 ^a^	24.03 ± 0.01 ^d^	28.33 ± 0.27 ^e^
p-Hidroxybenxoic ac.	n.d.	0.57 ± 0.13 ^a,b^	0.34 ± 0.37 ^a^	0.68 ± 0.03 ^a,b^	0.19 ± 0.19 ^a^	3.20 ± 0.06 ^c^	1.98 ± 0.12 ^b,c^	16.49 ± 0.68 ^d^	15.77 ± 0.80 ^d^
Syringic ac.	n.d.	2.61 ± 0.95 ^a,b^	2.55 ± 0.72 ^a,b^	2.91 ± 0.00 ^a,b^	1.98 ± 0.01 ^a^	3.70 ± 0.22 ^b,c^	3.30 ± 0.10 ^a,b^	5.22 ± 0.02 ^c,d^	6.69 ± 0.07 ^d^
Vanillic ac.	n.d.	3.79 ± 1.17 ^a^	2.22 ± 0.31 ^a^	2.98 ± 0.10 ^a^	1.82 ± 0.08 ^a^	3.21 ± 0.02 ^a^	2.83 ± 0.09 ^a^	6.40 ± 0.20 ^b^	6.62 ± 1.01^b^
Caffeic ac.	n.d.	0.63 ± 0.03 ^a^	0.60 ± 0.04 ^a^	n.d.	n.d.	n.d.	n.d.	n.q.	n.q.
Vanillin	n.d.	4.56 ± 0.32 ^b,c^	4.10 ± 0.56 ^b^	4.18 ± 0.01 ^b^	1.33 ± 0.03 ^a^	5.22 ± 0.06 ^c,d^	4.69 ± 0.04 ^b,c^	5.88 ± 0.02 ^d^	6.03 ± 0.00 ^d^
Coniferylaldehyde	n.d.	6.97 ± 0.65 ^b^	7.50 ± 0.91 ^b,c^	6.66 ± 0.03 ^b^	4.85 ± 0.02 ^a^	8.22 ± 0.06 ^b,c^	7.72 ± 0.01 ^b,c^	7.81 ± 0.03 ^b,c^	8.94 ± 0.00 ^c^
Sinapaldehyde	n.d.	11.73 ± 1.68 ^b^	13.73 ± 1.21 ^b^	11.32 ± 0.00 ^b^	8.39 ± 0.02 ^a^	19.02 ± 0.14 ^c^	16.91 ± 0.03 ^c^	19.05 ± 0.00 ^c^	24.25 ± 0.01 ^d^
Syringaldehyde	n.d.	12.49 ± 1.48 ^c^	10.67 ± 1.04 ^b,c^	9.62 ± 0.05 ^b^	6.00 ± 0.03 ^a^	11.19 ± 0.11 ^b,c^	10.20 ± 0.00 ^b,c^	10.04 ± 0.04 ^b,c^	11.57 ± 0.04 ^b,c^
Furfural	0.45 ± 0.01 ^a^	6.52 ± 0.26 ^d^	5.89 ± 0.11 ^c^	7.71 ± 0.02 ^e^	3.70 ± 0.01 ^b^	10.05 ± 0.05 ^f^	11.01 ± 0.02 ^f^	10.69 ± 0.18 ^g^	14.54 ± 0.07 ^h^
5-MF	n.d.	1.45 ± 0.29 ^b,c,d^	1.09 ± 0.16 ^a,b^	1.24 ± 0.01 ^b,c^	0.63 ± 0.02 ^a^	1.71 ± 0.05 ^c,d^	1.72 ± 0.02 ^c,d^	1.25 ± 0.06 ^b,c^	1.84 ± 0.07 ^d^
5-HMF	n.d.	4.31 ± 0.06 ^c^	3.64 ± 0.26 ^b^	4.02 ± 0.08 ^b,c^	2.21 ± 0.03 ^a^	5.50 ± 0.04 ^d^	5.54 ± 0.03 ^d^	5.11 ± 0.14 ^d^	7.68 ± 0.08 ^e^
TPI	n.d.	306.90 ± 2.6 ^b^	283.30 ± 0.0 ^a,b^	319.60 ± 3.90 ^b^	236.90 ± 1.30 ^a^	554.20 ± 1.30 ^c^	509.20 ± 0.60 ^c^	1072.80 ± 19.90 ^d^	1247.80 ± 28.30 ^e^

Data are mean value ± standard deviation; values in the same row with different letters are significantly different (*p* < 0.05). n.d.: Not detected.

**Table 3:** Chromatic characteristics of distillates ageing in barrel of American, French and Spanish oak, and Chestnut.

	Initial	American Oak		French Oak		Spanish Oak		Chestnut	
		Intense Toast	Medium Toast	Intense Toast	Medium Toast	Intense Toast	Medium Toast	Intense Toast	Medium Toast
Lightness (L*) %	93.2 ± 0.5 ^f^	90.7 ± 1.1 ^d,e^	92.4 ± 0.0 ^e,f^	90.2 ± 1.0 ^d^	92.4 ± 0.0 ^e,f^	84.1 ± 0.0 ^c^	85.5 ± 0.1 ^c^	72.9 ± 0.0 ^b^	65.7 ± 0.1 ^a^
Green-Red hues (a*)	0.04 ± 0.02 ^c,d^	0.21 ± 0.16 ^d^	−1.02 ± 0.08 ^a,b^	−0.56 ± 0.40 ^b,c^	−1.23 ± 0.00 ^a^	5.72 ± 0.09 ^f^	3.37 ± 0.06 ^e^	15.60 ± 0.10 ^g^	20.40 ± 0.00 ^h^
Blue-Yellow hues (b*)	2.13 ± 0.03 ^a^	37.2 ± 0.2 ^d^	34.3 ± 0.0 ^c^	34.2 ± 0.1 ^c^	29.1 ± 0.0 ^b^	59.2 ± 0.1 ^f^	54.5 ± 0.2 ^e^	72.4 ± 0.0 ^g^	76.2 ± 0.1 ^h^
CIEDE 2000 (ΔE_00_)		18.7 ± 0.0 ^c^	17.7 ± 0.0 ^b^	17.8 ± 0.0 ^b^	15.9 ± 0.0 ^a^	24.7 ± 0.0 ^e^	23.6 ± 0.0 ^d^	29.9 ± 0.0 ^f^	33.3 ± 0.1 ^g^

Data are mean value ± standard deviation; values in the same row with different letters are significantly different (*p* < 0.05). n.d.: Not detected; n.q.: Not quantified.

**Table 4:** Organic acid contents (mg/L) of distillates ageing in barrel of American, French and Spanish oak, and Chestnut.

	Initial	American Oak		French Oak		Spanish Oak		Chestnut	
		Intense Toast	Medium Toast	Intense Toast	Medium Toast	Intense Toast	Medium Toast	Intense Toast	Medium Toast
Succinic ac.	n.d.	30.1 ± 4.7 ^b^	25.9 ± 0.3 ^a,b^	27.6 ± 0.1 ^b^	19.2 ± 0.9 ^a^	27.2 ± 0.4 ^b^	25.0 ± 0.0 ^a,b^	31.7 ± 0.5 ^b^	28.4 ± 1.9 ^b^
Lactic ac.	181.6 ± 4.4 ^a^	403.8 ± 49.9 ^b^	367.5 ± 2.5 ^b^	338.9 ± 0.8 ^b^	333.5 ± 1.8 ^b^	175.2 ± 6.0 ^a^	173.6 ± 0.7 ^a^	188.7 ± 13.1 ^a^	180.2 ± 14.2 ^a^
Acetic ac.	127.6 ± 0.1 ^a^	307.6 ± 32.7 ^d^	265.6 ± 3.7 ^c,d^	266.2 ± 3.7 ^c,d^	247.9 ± 6.3 ^b,c^	227.9 ± 7.1 ^b,c^	210.1 ± 4.9 ^b^	243.7 ± 3.6 ^b,c^	259.1 ± 1.6 ^c^

Data are mean value ± standard deviation; values in the same row with different letters are significantly different (*p* < 0.05). n.d.: Not detected.

**Table 5:** Weights of the variables for each Principal Component of PCA.

	PC1 (74.8%)	PC2 (17.4%)	PC3 (5.6%)
Gallic ac.	0.212098	0.229747	0.405247
p-Hidroxybenxoic ac.	0.212616	0.285379	0.269082
Syringic ac.	0.260176	0.0537041	0.0704321
Vanillic ac.	0.249766	0.0422214	0.26319
Vanillin	0.248734	−0.124367	−0.108984
Coniferyaldehyde	0.235514	−0.228758	−0.129214
Sinapaldehyde	0.258133	−0.0318904	−0.161783
Syringaldehyde	0.210764	−0.324929	−0.0632737
Furfural	0.255016	0.0142459	−0.225365
5-MF	0.228023	−0.191001	−0.319289
5-HMF	0.254304	−0.0660727	−0.195005
TPI	0.248244	0.182906	0.104234
L*	−0.231347	−0.259389	−0.0834878
a*	0.219554	0.296211	0.120918
b*	0.259380	0.0432476	−0.107897
ΔE_00_	0.260317	−0.0367621	−0.040682
Succinic ac.	0.216806	−0.293651	0.0711698
Lactic ac.	−0.073157	−0.444863	0.491908
Acetic ac.	0.137467	−0.412107	0.383191

## Supplementary Materials

The following are available online at https://www.mdpi.com/2304-8158/9/3/250/s1, Table S1: Phenolic compounds contents (mg/L of absolute alcohol) and TPI (mg gallic acid equivalent by L of absolute alcohol) of distillates ageing in barrel of American, French and Spanish oak, and Chestnut.
