# Supplementary material for: Use of Alternative Wood for the Ageing of Brandy de Jerez
Source: Foods. 2020 Feb 26;9(3):250. doi: 10.3390/foods9030250 (PMC7143003; doi:10.3390/foods9030250)
Supplement: Supplementary file 1 [file foods-09-00250-s001.pdf]

Table S1. Phenolic compounds contents (mg/L of absolute alcohol) and TPI (mg gallic acid equivalent by L of absolute alcohol) of distillates ageing in barrel of American, French and Spanish oak, and Chestnut.

	Initial	American Oak		French Oak		Spanish Oak		Chestnut	
		Intense toast	Medium toast	Intense toast	Medium toast	Intense toast	Medium toast	Intense toast	Medium toast
Gallic ac.	n.d.	8.68 ± 0.18^{b}	8.35 ± 0.88^{b}	5.30 ± 0.20^{ab}	4.84 ± 0.11^{ab}	4.81 ± 0.04^{ab}	3.30 ± 0.45^{a}	43.37 ± 2.24^{c}	51.30 ± 2.18^{d}
p-Hidroxybenxoic ac.	n.d.	1.01 ± 0.19^{a}	0.61 ± 0.66^{a}	1.18 ± 0.01^{a}	0.32 ± 0.32^{a}	5.40 ± 0.11^{c}	3.28 ± 0.12^{b}	29.73 ± 0.32^{e}	28.53 ± 0.03^{d}
Syingic ac.	n.d.	4.61 ± 1.48^{a}	4.62 ± 1.21^{a}	5.07 ± 0.28^{a}	3.45 ± 0.14^{a}	6.24 ± 0.12^{a}	5.46 ± 0.03^{a}	9.42 ± 0.45^{b}	12.12 ± 0.50^{b}
Vanillic ac.	n.d.	6.70 ± 1.78^{b}	4.03 ± 0.48^{ab}	5.19 ± 0.11^{ab}	3.17 ± 0.00^{a}	5.42 ± 0.18^{ab}	4.68 ± 0.03^{ab}	11.54 ± 0.24^{c}	11.94 ± 1.21^{c}
Caffeic ac.	n.d.	1.12 ± 0.00^{a}	1.09 ± 0.05^{a}	0.17 ± 0.03	n.d.	n.d.	n.d.	0.29 ± 0.00	0.45 ± 0.01
Vanillin	n.d.	8.10 ± 0.20^{b}	7.44 ± 0.86^{b}	7.29 ± 0.38^{b}	2.32 ± 0.05^{a}	8.81 ± 0.25^{b}	7.76 ± 0.13^{b}	10.61 ± 0.52^{c}	10.92 ± 0.57^{c}
Coniferylaldehyde	n.d.	12.38 ± 0.60^{b}	13.61 ± 1.37^{bc}	11.61 ± 0.58^{b}	8.45 ± 0.34^{a}	13.87 ± 0.45^{bc}	12.78 ± 0.30^{b}	14.09 ± 0.68^{bc}	16.19 ± 0.84^{c}
Sinapaldehyde	n.d.	20.81 ± 2.05^{b}	24.93 ± 1.67^{bc}	19.73 ± 1.08^{ab}	14.62 ± 0.62^{a}	32.09 ± 1.05^{ce}	28.00 ± 0.65^{cd}	34.38 ± 1.79^{e}	43.93 ± 2.26f
Syringaldehyde	n.d.	22.17 ± 1.63^{d}	19.37 ± 1.48^{bcd}	16.77 ± 0.83^{b}	10.45 ± 0.41^{a}	18.88 ± 0.57^{bcd}	16.89 ± 0.42^{b}	18.12 ± 0.87^{bc}	20.96 ± 1.02^{cd}
Furfural	0.73 ± 0.01^{a}	11.59 ± 0.06^{cd}	10.70 ± 0.03^{c}	13.44 ± 0.70^{d}	6.45 ± 0.27^{b}	16.96 ± 0.59^{e}	18.23 ± 0.43^{e}	19.29 ± 0.68^{e}	26.33 ± 1.24f
5-MF	n.d.	2.57 ± 0.40^{bc}	1.98 ± 0.25^{b}	2.16 ± 0.10^{b}	1.10 ± 0.01^{a}	2.88 ± 0.03^{cd}	2.85 ± 0.04^{cd}	2.25 ± 0.01^{bc}	3.33 ± 0.05^{d}
5-HMF	n.d.	7.67 ± 0.24^{b}	6.61 ± 0.33^{b}	7.00 ± 0.24^{b}	3.85 ± 0.12^{a}	9.28 ± 0.30^{c}	9.17 ± 0.18^{c}	9.22 ± 0.23^{c}	13.91 ± 0.58^{d}
TPI	n.d..	546.1 ± 20.0^{a}	514.8 ± 10.8^{a}	557.0 ± 23.8^{a}	412.7 ± 16.1^{a}	935.1 ± 35.2^{b}	843.0 ± 20.1^{b}	1935.4 ± 64.9^{c}	2259.0 ± 66.1^{d}
